# Methyl 3-(2-furyl­methyl­idene)carbazate

**DOI:** 10.1107/S1600536810051019

**Published:** 2010-12-11

**Authors:** Yu-Feng Li

**Affiliations:** a Microscale Science Institute, Department of Chemistry and Chemical Engineering, Weifang University, Weifang 261061, People’s Republic of China

## Abstract

The asymmetric unit of the title compound, C_7_H_8_N_2_O_3_, contains two approximately planar mol­ecules (r.m.s. deviations = 0.058 and 0.070 Å). In the crystal, mol­ecules are linked into [010] chains by way of alternating N—H⋯O and N—H⋯(N,O) hydrogen-bond linkages.

## Related literature

For a related structure, see: Li & Jian (2010 ▶).
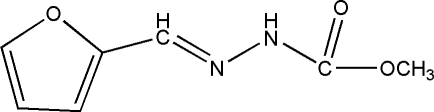


## Experimental

### 

#### Crystal data

C_7_H_8_N_2_O_3_
*M* *_r_* = 168.15
Monoclinic, 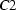

*a* = 14.668 (5) Å
*b* = 7.7356 (15) Å
*c* = 14.720 (3) Å
β = 104.11 (4)°
*V* = 1619.8 (7) Å^3^
*Z* = 8
Mo *K*α radiation
μ = 0.11 mm^−1^
*T* = 293 K
0.25 × 0.22 × 0.18 mm

#### Data collection

Bruker SMART CCD diffractometer
7823 measured reflections
3695 independent reflections
2600 reflections with *I* > 2σ(*I*)
*R*_int_ = 0.117

#### Refinement

*R*[*F* ^2^ > 2σ(*F* ^2^)] = 0.072
*wR*(*F* ^2^) = 0.186
*S* = 0.89
3695 reflections
217 parameters
1 restraint
H-atom parameters constrained
Δρ_max_ = 0.37 e Å^−3^
Δρ_min_ = −0.28 e Å^−3^

### 

Data collection: *SMART* (Bruker, 1997 ▶); cell refinement: *SAINT* (Bruker, 1997 ▶); data reduction: *SAINT*; program(s) used to solve structure: *SHELXS97* (Sheldrick, 2008 ▶); program(s) used to refine structure: *SHELXL97* (Sheldrick, 2008 ▶); molecular graphics: *SHELXTL* (Sheldrick, 2008 ▶); software used to prepare material for publication: *SHELXTL*.

## Supplementary Material

Crystal structure: contains datablocks global, I. DOI: 10.1107/S1600536810051019/hb5763sup1.cif

Structure factors: contains datablocks I. DOI: 10.1107/S1600536810051019/hb5763Isup2.hkl

Additional supplementary materials:  crystallographic information; 3D view; checkCIF report

## Figures and Tables

**Table 1:** Hydrogen-bond geometry (Å, °)

*D*—H⋯*A*	*D*—H	H⋯*A*	*D*⋯*A*	*D*—H⋯*A*
N1—H1*A*⋯O5^i^	0.86	2.07	2.867 (4)	155
N3—H3*A*⋯O2	0.86	2.30	3.085 (4)	152
N3—H3*A*⋯N2	0.86	2.53	3.232 (3)	140

Symmetry code: (i) 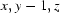.

**Fig. 1.**
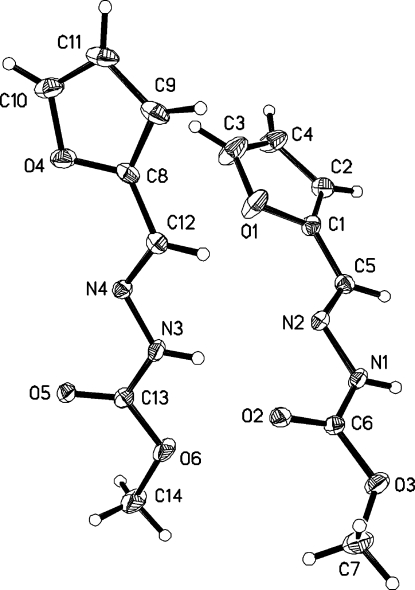
The structure of the title compound showing 30% probability displacement ellipsoids.

## supplementary crystallographic information

### Experimental

A mixture of methyl carbazate (0.1 mol), and furfural (0.1 mol) was stirred in refluxing ethanol (20 mL) for 4 h to afford the title compound (0.085 mol, yield 85%).  Colourless blocks of the title compound were obtained by recrystallization from ethanol at room temperature.

### Refinement

The absolute structure was indeterminate in the present study. H atoms were fixed geometrically and allowed to ride on their attached atoms, with C—H distances = 0.93–0.97 Å; N—H = 0.86Å and with *U*_iso_(H) = 1.2*U*_eq_(C,*N*) or 1.5*U*_eq_(C_methyl_).

### Crystal data

**Table:**

C_7_H_8_N_2_O_3_	*F*(000) = 704
*M**_r_* = 168.15	*D*_x_ = 1.379 Mg m^−^^3^
Monoclinic, *C*2	Mo *K*α radiation, λ = 0.71073 Å
*a* = 14.668 (5) Å	Cell parameters from 3695 reflections
*b* = 7.7356 (15) Å	θ = 3.2–27.5°
*c* = 14.720 (3) Å	µ = 0.11 mm^−^^1^
β = 104.11 (4)°	*T* = 293 K
*V* = 1619.8 (7) Å^3^	Block, colorless
*Z* = 8	0.25 × 0.22 × 0.18 mm

### Data collection

**Table:**

Bruker SMART CCD diffractometer	2600 reflections with *I* > 2σ(*I*)
Radiation source: fine-focus sealed tube	*R*_int_ = 0.117
graphite	θ_max_ = 27.5°, θ_min_ = 3.2°
phi and ω scans	*h* = −16→18
7823 measured reflections	*k* = −9→10
3695 independent reflections	*l* = −19→19

### Refinement

**Table:**

Refinement on *F*^2^	Primary atom site location: structure-invariant direct methods
Least-squares matrix: full	Secondary atom site location: difference Fourier map
*R*[*F*^2^ > 2σ(*F*^2^)] = 0.072	Hydrogen site location: inferred from neighbouring sites
*wR*(*F*^2^) = 0.186	H-atom parameters constrained
*S* = 0.89	*w* = 1/[σ^2^(*F*_o_^2^) + (0.1*P*)^2^] where *P* = (*F*_o_^2^ + 2*F*_c_^2^)/3
3695 reflections	(Δ/σ)_max_ < 0.001
217 parameters	Δρ_max_ = 0.37 e Å^−^^3^
1 restraint	Δρ_min_ = −0.28 e Å^−^^3^

### Special details

**Table:**

Geometry. All e.s.d.'s (except the e.s.d. in the dihedral angle between two l.s. planes) are estimated using the full covariance matrix. The cell e.s.d.'s are taken into account individually in the estimation of e.s.d.'s in distances, angles and torsion angles; correlations between e.s.d.'s in cell parameters are only used when they are defined by crystal symmetry. An approximate (isotropic) treatment of cell e.s.d.'s is used for estimating e.s.d.'s involving l.s. planes.
Refinement. Refinement of *F*^2^ against ALL reflections. The weighted *R*-factor *wR* and goodness of fit *S* are based on *F*^2^, conventional *R*-factors *R* are based on *F*, with *F* set to zero for negative *F*^2^. The threshold expression of *F*^2^ > σ(*F*^2^) is used only for calculating *R*-factors(gt) *etc*. and is not relevant to the choice of reflections for refinement. *R*-factors based on *F*^2^ are statistically about twice as large as those based on *F*, and *R*- factors based on ALL data will be even larger.

### Fractional atomic coordinates and isotropic or equivalent isotropic displacement parameters (Å^2^)

**Table:**

	*x*	*y*	*z*	*U*_iso_*/*U*_eq_	
O5	0.18498 (16)	0.9708 (3)	0.08083 (14)	0.0286 (5)	
N4	0.22925 (19)	0.8558 (3)	0.25908 (17)	0.0242 (5)	
N3	0.1797 (2)	0.7585 (3)	0.18594 (17)	0.0281 (6)	
H3A	0.1595	0.6572	0.1951	0.034*	
O4	0.33569 (18)	1.0262 (3)	0.41175 (15)	0.0358 (6)	
O6	0.1200 (2)	0.7115 (3)	0.03597 (15)	0.0397 (7)	
C8	0.2972 (2)	0.8690 (4)	0.4211 (2)	0.0276 (7)	
C12	0.2438 (2)	0.7836 (4)	0.3391 (2)	0.0260 (7)	
H12A	0.2194	0.6741	0.3443	0.031*	
C13	0.1635 (2)	0.8259 (4)	0.0994 (2)	0.0261 (7)	
C9	0.3199 (3)	0.8194 (5)	0.5124 (2)	0.0376 (9)	
H9A	0.3020	0.7180	0.5371	0.045*	
C14	0.0973 (4)	0.7748 (5)	−0.0591 (2)	0.0489 (11)	
H14A	0.0666	0.6853	−0.1005	0.073*	
H14B	0.1539	0.8086	−0.0759	0.073*	
H14C	0.0562	0.8728	−0.0640	0.073*	
C11	0.3764 (3)	0.9529 (6)	0.5622 (2)	0.0419 (9)	
H11A	0.4032	0.9564	0.6263	0.050*	
C10	0.3839 (3)	1.0730 (6)	0.4994 (2)	0.0421 (9)	
H10A	0.4176	1.1754	0.5137	0.051*	
O2	0.04032 (17)	0.4665 (3)	0.19917 (14)	0.0296 (5)	
N1	0.1456 (2)	0.2564 (4)	0.18947 (19)	0.0291 (6)	
H1A	0.1539	0.1531	0.1715	0.035*	
N2	0.22041 (19)	0.3544 (3)	0.23547 (17)	0.0265 (6)	
C6	0.0590 (2)	0.3252 (4)	0.1731 (2)	0.0272 (7)	
O3	−0.00370 (18)	0.2153 (3)	0.12255 (17)	0.0371 (6)	
C5	0.3004 (2)	0.2814 (4)	0.2473 (2)	0.0264 (7)	
H5A	0.3037	0.1687	0.2264	0.032*	
O1	0.37821 (19)	0.5284 (3)	0.3290 (2)	0.0509 (8)	
C1	0.3852 (2)	0.3697 (4)	0.2920 (2)	0.0282 (7)	
C2	0.4759 (3)	0.3280 (5)	0.3040 (2)	0.0348 (8)	
H2B	0.4995	0.2257	0.2857	0.042*	
C7	−0.0995 (3)	0.2726 (6)	0.0996 (3)	0.0501 (10)	
H7A	−0.1386	0.1857	0.0629	0.075*	
H7B	−0.1046	0.3780	0.0644	0.075*	
H7C	−0.1194	0.2924	0.1562	0.075*	
C4	0.5291 (3)	0.4693 (6)	0.3499 (3)	0.0463 (10)	
H4B	0.5943	0.4781	0.3669	0.056*	
C3	0.4682 (3)	0.5862 (6)	0.3638 (3)	0.0587 (13)	
H3B	0.4842	0.6924	0.3929	0.070*	

### Atomic displacement parameters (Å^2^)

**Table:**

	*U*^11^	*U*^22^	*U*^33^	*U*^12^	*U*^13^	*U*^23^
O5	0.0362 (13)	0.0239 (12)	0.0235 (10)	−0.0029 (10)	0.0033 (9)	−0.0002 (8)
N4	0.0261 (13)	0.0244 (14)	0.0213 (12)	0.0009 (11)	0.0044 (9)	−0.0031 (10)
N3	0.0392 (16)	0.0191 (12)	0.0258 (13)	−0.0054 (11)	0.0074 (11)	−0.0036 (10)
O4	0.0394 (15)	0.0431 (15)	0.0218 (11)	−0.0052 (11)	0.0018 (10)	0.0004 (9)
O6	0.0650 (19)	0.0328 (14)	0.0197 (11)	−0.0137 (12)	0.0070 (11)	−0.0089 (9)
C8	0.0252 (17)	0.033 (2)	0.0244 (15)	0.0089 (13)	0.0056 (12)	0.0049 (12)
C12	0.0236 (15)	0.0287 (17)	0.0274 (15)	0.0047 (12)	0.0093 (12)	0.0010 (12)
C13	0.0276 (16)	0.0272 (17)	0.0249 (15)	−0.0004 (13)	0.0090 (12)	−0.0056 (12)
C9	0.040 (2)	0.049 (2)	0.0234 (16)	0.0136 (17)	0.0078 (14)	0.0077 (14)
C14	0.078 (3)	0.046 (2)	0.0189 (16)	−0.018 (2)	0.0044 (17)	−0.0067 (15)
C11	0.038 (2)	0.064 (3)	0.0198 (15)	0.0132 (18)	0.0010 (14)	−0.0020 (16)
C10	0.044 (2)	0.052 (2)	0.0260 (17)	−0.0013 (18)	0.0013 (15)	−0.0107 (16)
O2	0.0335 (12)	0.0265 (12)	0.0247 (11)	0.0025 (10)	−0.0006 (9)	−0.0045 (9)
N1	0.0292 (15)	0.0200 (13)	0.0360 (14)	−0.0038 (11)	0.0039 (11)	−0.0066 (10)
N2	0.0294 (15)	0.0209 (13)	0.0273 (13)	−0.0028 (11)	0.0035 (10)	−0.0039 (10)
C6	0.0319 (18)	0.0264 (16)	0.0206 (14)	−0.0028 (13)	0.0015 (12)	−0.0025 (12)
O3	0.0319 (14)	0.0315 (14)	0.0434 (14)	−0.0036 (10)	0.0006 (11)	−0.0118 (10)
C5	0.0294 (17)	0.0275 (16)	0.0223 (14)	0.0021 (12)	0.0062 (12)	−0.0001 (11)
O1	0.0242 (14)	0.0374 (15)	0.088 (2)	−0.0019 (11)	0.0086 (13)	−0.0304 (14)
C1	0.0311 (18)	0.0256 (17)	0.0284 (16)	0.0014 (13)	0.0082 (13)	−0.0049 (12)
C2	0.0346 (19)	0.0350 (18)	0.0327 (17)	0.0130 (15)	0.0043 (14)	−0.0072 (14)
C7	0.033 (2)	0.051 (2)	0.058 (2)	−0.0012 (18)	−0.0055 (17)	−0.016 (2)
C4	0.0242 (19)	0.055 (2)	0.055 (2)	0.0022 (17)	0.0021 (16)	−0.023 (2)
C3	0.025 (2)	0.054 (3)	0.093 (3)	−0.0031 (18)	0.006 (2)	−0.035 (2)

### Geometric parameters (Å, °)

**Table:**

O5—C13	1.214 (4)	O2—C6	1.211 (4)
N4—C12	1.273 (4)	N1—C6	1.344 (4)
N4—N3	1.368 (3)	N1—N2	1.369 (4)
N3—C13	1.343 (4)	N1—H1A	0.8600
N3—H3A	0.8600	N2—C5	1.275 (4)
O4—C10	1.360 (4)	C6—O3	1.338 (4)
O4—C8	1.361 (4)	O3—C7	1.432 (5)
O6—C13	1.331 (4)	C5—C1	1.431 (4)
O6—C14	1.442 (4)	C5—H5A	0.9300
C8—C9	1.359 (4)	O1—C1	1.357 (4)
C8—C12	1.430 (4)	O1—C3	1.370 (5)
C12—H12A	0.9300	C1—C2	1.339 (5)
C9—C11	1.412 (6)	C2—C4	1.414 (6)
C9—H9A	0.9300	C2—H2B	0.9300
C14—H14A	0.9600	C7—H7A	0.9600
C14—H14B	0.9600	C7—H7B	0.9600
C14—H14C	0.9600	C7—H7C	0.9600
C11—C10	1.334 (6)	C4—C3	1.321 (6)
C11—H11A	0.9300	C4—H4B	0.9300
C10—H10A	0.9300	C3—H3B	0.9300
			
C12—N4—N3	115.1 (3)	C6—N1—N2	118.5 (3)
C13—N3—N4	118.1 (3)	C6—N1—H1A	120.7
C13—N3—H3A	121.0	N2—N1—H1A	120.7
N4—N3—H3A	121.0	C5—N2—N1	115.0 (3)
C10—O4—C8	105.9 (3)	O2—C6—O3	124.8 (3)
C13—O6—C14	114.4 (3)	O2—C6—N1	125.3 (3)
C9—C8—O4	110.1 (3)	O3—C6—N1	109.9 (3)
C9—C8—C12	131.0 (3)	C6—O3—C7	115.9 (3)
O4—C8—C12	118.9 (3)	N2—C5—C1	121.2 (3)
N4—C12—C8	120.8 (3)	N2—C5—H5A	119.4
N4—C12—H12A	119.6	C1—C5—H5A	119.4
C8—C12—H12A	119.6	C1—O1—C3	106.6 (3)
O5—C13—O6	124.2 (3)	C2—C1—O1	109.6 (3)
O5—C13—N3	125.2 (3)	C2—C1—C5	132.1 (3)
O6—C13—N3	110.6 (3)	O1—C1—C5	118.4 (3)
C8—C9—C11	106.2 (3)	C1—C2—C4	106.9 (3)
C8—C9—H9A	126.9	C1—C2—H2B	126.5
C11—C9—H9A	126.9	C4—C2—H2B	126.5
O6—C14—H14A	109.5	O3—C7—H7A	109.5
O6—C14—H14B	109.5	O3—C7—H7B	109.5
H14A—C14—H14B	109.5	H7A—C7—H7B	109.5
O6—C14—H14C	109.5	O3—C7—H7C	109.5
H14A—C14—H14C	109.5	H7A—C7—H7C	109.5
H14B—C14—H14C	109.5	H7B—C7—H7C	109.5
C10—C11—C9	106.6 (3)	C3—C4—C2	106.8 (3)
C10—C11—H11A	126.7	C3—C4—H4B	126.6
C9—C11—H11A	126.7	C2—C4—H4B	126.6
C11—C10—O4	111.1 (4)	C4—C3—O1	110.2 (4)
C11—C10—H10A	124.4	C4—C3—H3B	124.9
O4—C10—H10A	124.4	O1—C3—H3B	124.9

### Hydrogen-bond geometry (Å, °)

**Table:**

*D*—H···*A*	*D*—H	H···*A*	*D*···*A*	*D*—H···*A*
N1—H1A···O5^i^	0.86	2.07	2.867 (4)	155
N3—H3A···O2	0.86	2.30	3.085 (4)	152
N3—H3A···N2	0.86	2.53	3.232 (3)	140

Symmetry codes: (i) *x*, *y*−1, *z*.
